# A sarcopenia screening test predicts mortality in hospitalized older adults

**DOI:** 10.1038/s41598-018-21237-9

**Published:** 2018-02-13

**Authors:** Tianjiao Tang, Linna Wu, Ling Yang, Jiaojiao Jiang, Qiukui Hao, Birong Dong, Ming Yang

**Affiliations:** 1 The Center of Gerontology and Geriatrics, West China Hospital, Sichuan University, No.37 Guoxue Lane, Chengdu, Sichuan China; 2 Health Management Center, West China Hospital, Sichuan University, No.37 Guoxue Lane, Chengdu, Sichuan China; 3 Outpatient Department, West China Hospital, Sichuan University, No.37 Guoxue Lane, Chengdu, Sichuan China; 4 The Center of Rehabilitation, West China Hospital, Sichuan University, No.37 Guoxue Lane, Chengdu, Sichuan China

## Abstract

The aim of this study is to investigate the validation of a sarcopenia screening test (Ishii’s formula) for predicting long-term mortality among older adult inpatients. A prospective, observational study was conducted in acute geriatric wards at three hospitals in western China. Sarcopenia was estimated using Ishii’s formula. Survival status was assessed at 12, 24, and 36 months after the baseline investigation. Cox proportional-hazard models were applied to calculate the hazard ratio for mortality associated with sarcopenia. Three hundred and eighty participants (100 women) with a mean age of 80.2 ± 7.1 years were included. According to Ishii’s formula, 264 participants (69.5%) were sarcopenic. The prevalence of sarcopenia was similar in men and women (71.1% vs. 65.0%, respectively, P = 0.258). Sixty-seven participants (17.6%) died during the 3-year follow-up period. The all-cause mortality was significantly higher in the sarcopenia group than in the non-sarcopenia group (20.1% vs. 12.1%, respectively, P < 0.05). Multivariate Cox proportional hazards analysis identified sarcopenia as a significant predictor of 3-year all-cause mortality (adjusted hazard ratio [HR]: 2.06; 95% confidence interval [CI]: 1.02–4.15). In conclusion, sarcopenia, estimated by Ishii’s formula, can predict 3-year all-cause mortality in a study population of hospitalized older adults.

## Introduction

Sarcopenia is a geriatric syndrome characterized by a loss of muscle mass, strength, and function^1^. It is prevalent in older adults, especially in those who are hospitalized or institutionalized^2^. Recently, sarcopenia was given an international classification of disease, tenth revision, clinical modification (ICD-10-CM) code, which represented “a major step forward” in understanding it as a disease^3^.

Sarcopenia has been associated with an increased risk of many adverse events, such as functional decline, falls, disability, poor quality of life and increased mortality^4–7^. Early interventions, such as resistance exercise and nutritional supplements, can effectively slow the progression of sarcopenia and prevent physical disability^8–10^. However, sarcopenia is usually not noticeable in its early phase until a critical event, such as a fall has occurred or physical disability has set in. Therefore, it is important to raise public awareness of sarcopenia and implement a validated screening test to detect sarcopenia in older adults as early as possible.

Several screening tools for sarcopenia have been developed. For example, the SARC-F questionnaire, the first screening tool, has been validated in different populations^11,12^. Recently, Shinya Ishii and his colleagues^13^ developed a new, simple screening test for sarcopenia (named Ishii’s formula). Unlike the SARC-F, Ishii’s formula was only based on age, calf circumference (CC) and handgrip strength (HS), which are easy to perform in clinical practice. It had reasonable sensitivity and specificity with a cut-point of 105 for older women (Sensitivity 75.5%, Specificity 92.0%) and 120 for older men (Sensitivity 84.9%, Specificity 88.2%). These features make it a potentially useful screening tool for identifying sarcopenia.

Ishii’s formula was developed using a sample of Japanese older adults and it has not been widely validated in other ethnic groups. In addition, it is unclear whether this tool is valuable for predicting the prognosis of hospitalized older adults. We, therefore, conducted a prospective study to investigate the prevalence of sarcopenia defined using Ishii’s formula and to evaluate whether this estimation of sarcopenia can predict long-term mortality among a study population of hospitalized older adults.

## Methods

This prospective study was conducted in the acute geriatric wards of three hospitals located in Chengdu, China: West China Hospital of Sichuan University, the Fifth People’s Hospital of Chengdu City and Sichuan Provincial Rehabilitation Hospital. The study protocol was approved by the Research Ethics Committee of Sichuan University. Written informed consent was obtained from all participants or their legal proxies. All methods in this study were in accordance with relevant regulations and guidelines.

### Participants in the baseline investigation

Between February and August 2012, we recruited consecutively admitted patients aged 60 years and older. Patients with the following conditions at admission were excluded: 1) severe cognitive impairment; 2) delirium; and 3) clinically visible edema. Patients with missing data were also excluded from the analyses.

### Data collection

Through face-to-face interviews, well-trained interviewers obtained baseline data from all participants within 48 hours of admission. All interviewers received specific training and passed a training test before the formal study began. Then, trained staff performed the anthropometric measurements to collect the data for body weight, height, and CC.

### Sarcopenia screening

In this study, we adopted Ishii’s formula^13^ to calculate the sarcopenia scores. The formula to calculate the total scores were as follows: score in men, 0.62 × (age − 64) − 3.09 × (HS − 50) − 4.64 × (CC − 42); score in women, 0.80 × (age − 64) − 5.09 × (HS − 34) − 3.28 × (CC − 42). The cut-points for defining sarcopenia were ≥105 for men and ≥120 for women.

Trained nurses measured the CC by using a millimeter-graded tape measure around the largest part of the calf to the nearest 0.1 cm, with the participants placed in the supine position, with the left knee raised and the calf placed at a right angle to the thigh^14^.

The HS was measured by trained technicians using a handheld dynamometer based on strain gauge sensors (EH101, Xiangshan Inc., Guangdong, China) to the nearest 0.1 kg. Trained nurses measured the HS while the participants seated with the elbow flexed at a 110° angle, the wrist in a neutral position, and the interphalangeal joint of the index finger at a 90° angle. Both hands were measured three times and the highest value of either hand was recorded^15^.

### Gait speed

A walking test was performed to evaluate the gait speed (GS). Trained nurses asked the participants to walk a 4-meter course with a usual gait speed and recorded the consuming time. Gait speed was calculated using the equation: gait speed (m/s) = 4/consuming time. The participants were allowed to use canes or walkers during the walking test, if necessary^16^.

### Covariates

Based on previous studies, we collected the following covariates from the hospital information systems and the face-to-face interviews: sex, smoking status, alcohol drinking status, polypharmacy (defined as the concomitant use of five or more medications), and comorbidities (hypertension, ischemic heart disease, chronic obstructive pulmonary disease [COPD], diabetes, stroke, chronic kidney disease, acute infection, osteoarthritis, tumor of any type, gastrointestinal disease, liver disease, falls in the previous year, urinary incontinence, chronic pain). We also evaluated the participants’ nutritional status, cognitive function, and for depression using the revised version of Mini Nutritional Assessment short-form (MNA-SF)^17^, the Chinese version of the Mini-Mental Status Examination (MMSE)^18^, and the Chinese version of the 30-item Geriatric Depression Scale (GDS-30)^19^, respectively. The highest score for the MNA-SF is 14; a score between 8 and 11 indicates that the participant is ‘at risk of malnutrition’; and a score of ≤7 indicates that the participant has malnutrition^17^. Cognitive impairment is defined as an MMSE score ≤17 for patients who are illiterates, ≤20 for primary school graduates, and ≤24 for high school graduates or individuals with higher education^18^. A GDS-30 score of ≥11 suggests depression^19^. In addition, hemoglobin and prealbumin were obtained for each participant. Further, because previous studies addressed the sex difference of sarcopenia^20,21^, we also performed a subgroup analysis according to sex.

### Follow-up

At 12, 24, and 36 months during the 3-year follow-up period, we obtained the survival status of the participants via telephone interviews. All the death events were confirmed by using the Local Death Registry Database. The period from the first investigation to the date of death was recorded for the participants who died during the follow-up. The period from the first investigation to the end of the last follow-up was recorded for the participants who did not die.

### Statistical analysis

The descriptive variables are presented as absolute numbers and frequencies for categorical variables, as the mean ± standard deviation (SD) for normally distributed continuous variables, and as the median ± interquartile range (IQR) for non-normally distributed continuous variables. The Pearson chi-squared test and a one-way ANOVA were used for categorical variables and continuous variables, respectively. Data analyses were performed using SPSS version 20.0 (SPSS Inc., Chicago, IL, USA), with p < 0.05 indicating statistical significance.

We applied univariate and multivariate Cox proportional-hazard models to calculate the hazard ratio (HR) and 95% confidence interval (CI) for mortality associated with sarcopenia. Significant covariates identified in the univariate Cox models (P < 0.10) were included in the multivariate Cox model. In addition, survival curves were estimated using the Kaplan-Meier method and were compared using log-rank tests.

## Results

### Characteristics of the study population

**Figure 1:**
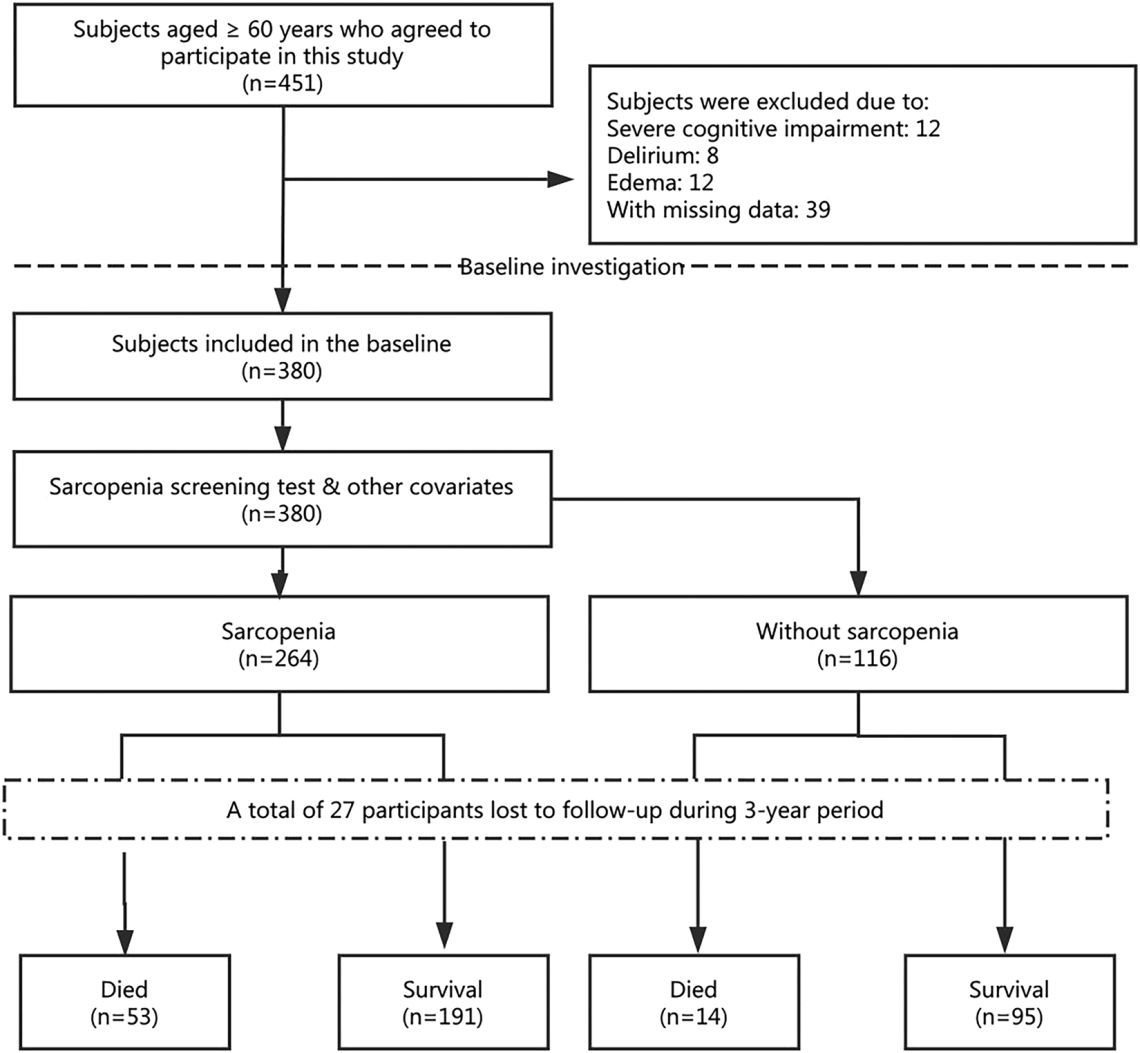
Study profile using the simple sarcopenia screening test (Ishii’s formula).

A total of 451 participants agreed to participate in the baseline investigation; 71 of them were excluded from the study because of severe cognitive impairment (12 individuals), delirium (8 individuals), edema (12 individuals), or with missing data (39 individuals). As a result, 380 participants (100 women, 280 men; mean age: 80.2 ± 7.1 years) were included in the baseline analyses. Twenty-seven participants were lost to follow-up during the 3-year period, resulting in a final sample size of 353 participants (Fig. 1). There was no significant difference between subjects who completed this study and those who lost to follow-up with regard to the baseline characteristics (Supplementary Table 1).

Table 1 shows the baseline characteristics of participants according to sarcopenia status. Age and common comorbidities were similar between the sarcopenia group and the non-sarcopenia group, except for COPD and stroke. Compared with the non-sarcopenia group, the sarcopenia group was more likely to have malnutrition (12.1% vs. 6.9%, respectively, P < 0.001) and cognitive impairment (41.1% vs. 21.0%, respectively, P < 0.001). In addition, both sarcopenic men and women exhibited a significantly lower BMI and GS compared with their non-sarcopenic counterparts. In addition, the HS and CC were also significantly lower in the sarcopenia group than in the non-sarcopenic group.

**Table 1:** Baseline characteristics of participants according to sarcopenia status.

Characteristic	No sarcopenia (n = 116)	Sarcopenia (n = 264)	p
Age (years)	79.7 ± 7.2	81.3 ± 6.9	0.535
Women	35 (30.2)	65 (24.6)	0.258
Current smokers	17 (14.7)	32 (12.1)	0.497
Current alcohol drinkers	19 (16.4)	27 (10.2)	0.090
Comorbidities			
Hypertension	70 (60.3)	155 (58.7)	0.766
Ischemic heart disease	44 (37.9)	79 (29.9)	0.124
COPD	25 (21.6)	94 (35.6)	0.007
Diabetes	35 (30.5)	69 (26.1)	0.416
Stroke	2 (1.7)	23 (8.7)	0.011
CKD	15 (12.9)	38 (14.4)	0.705
Acute infection	40 (34.5)	75 (28.4)	0.235
Osteoarthritis	25 (21.6)	78 (29.5)	0.106
Tumor of any type	8 (6.9)	34 (12.9)	0.087
GI disease	20 (17.2)	53 (20.1)	0.518
Liver disease	12 (10.3)	19 (7.2)	0.302
Falls in the previous year	9 (7.8)	37 (14.0)	0.085
Urinary incontinence	12 (10.3)	36 (13.6)	0.374
Chronic pain	35 (30.2)	84 (31.8)	0.750
Malnutrition	8 (6.9)	32 (12.1)	<0.001
Polypharmacy^*^	35 (43.2)	93 (48.4)	0.429
Cognitive impairment^*^	17 (21.0)	79 (41.1)	0.001
Depression^*^	18 (22.2)	50 (26.0)	0.505
BMI (kg/m^2^)			
Women	23.9 ± 3.6	22.1 ± 4.4	0.048
Men	23.7 ± 3.6	21.7 ± 3.6	<0.001
CC (cm)			
Women	34.4 ± 3.4	30.1 ± 4.0	<0.001
Men	35.5 ± 2.8	31.6 ± 3.5	<0.001
Gait speed (m/s)			
Women	0.8 ± 0.3	0.7 ± 0.2	0.027
Men	0.9 ± 0.5	0.7 ± 0.3	0.002
Handgrip strength (kg)			
Women	21.0 ± 3.8	10.2 ± 5.2	<0.001
Men	30.4 ± 6.4	18.8 ± 6.9	<0.001
Hemoglobin (g/L)	125.1 ± 21.1	121.3 ± 22.8	0.126
Prealbumin (mg/L)	202.0 ± 53.1	192.2 ± 65.7	0.291
1-year mortality	9 (8.3)	28 (11.5)	0.362
2-year mortality	10 (9.2)	43 (17.6)	0.040
3-year mortality	14 (12.8)	53 (21.7)	0.049

^*^The sample size was 273 due to missing data.

Data are presented as the number (percent) for the following variables: women, current smokers, current alcohol drinkers, and specific comorbidities listed above. For other variables, the mean ± SD are presented.

One-way ANOVA was used for the continuous variables, and the Pearson chi-squared test was used for categorical variables. During analyses, p < 0.05 was considered statistically significant.

BMI: body mass index; CC: calf circumference; CKD: chronic kidney disease; COPD: chronic obstructive pulmonary disease; GI: gastrointestinal

### Prevalence of sarcopenia

Based on the simple screening test, 264 participants (69.5%) suffered from sarcopenia. The prevalence of sarcopenia was similar in men and women (71.1% vs. 65.0%, respectively; P = 0.258).

### Association between sarcopenia and mortality

During the 3 years of follow-up, a total of 67 participants (17.6%) died, 53 of them were in the sarcopenia group. In addition, 27 participants (7.1%) lost to follow-up.

The 2-year and 3-year all-cause mortalities were significantly higher in the sarcopenia group than in the non-sarcopenia group, respectively (2-year mortality: 17.6 vs. 9.2%, p = 0.040; 3-year mortality: 21.7% vs. 12.8%, P = 0.049, respectively). The 1-year all-cause mortality was also higher in the sarcopenia group than in the non-sarcopenia group, but the result was not statistically significant (11.5% vs. 8.3%, p = 0.362).

Table 2 shows the results of the univariate and multivariate Cox proportional hazard analyses for 3-year all-cause mortality in the whole study population. The univariate analysis indicated that sarcopenic individuals were more likely to die compared with those without sarcopenia, but the difference was not statistically significant (hazard ratio [HR]: 1.77; 95% confidential interval [CI]: 0.98–3.19).

**Table 2:** Results of univariate and multivariate Cox proportional hazard analyses for 3-year all-cause mortality in the whole study population.

	Univariate analysis			Multivariate analysis		
	HR	95% CI	p	HR	95% CI	p
Age (years)	1.07	(1.03–1.12)	<0.001	Not included		
Women	0.55	(0.29–1.05)	0.069	Not included		
Sarcopenia	1.77	(0.98–3.19)	0.058	2.06	(1.02–4.15)	0.044
Current smokers	1.06	(0.51–2.22)	0.879	Not selected		
Current alcohol drinkers	1.38	(0.60–3.19)	0.454	Not selected		
Hypertension	1.24	(0.77–2.01)	0.370	Not selected		
Ischemic heart disease	1.30	(0.79–2.12)	0.298	Not selected		
COPD	1.10	(0.66–1.83)	0.704	Not selected		
Diabetes	1.19	(0.66–1.89)	0.673	Not selected		
Stroke	1.35	(0.45–4.31)	0.609	Not selected		
CKD	1.59	(0.87–2.91)	0.134	Not selected		
Acute infection	1.31	(1.03–4.89)	0.043	Not selected		
Osteoarthritis	0.88	(0.51–1.54)	0.672	Not selected		
Tumor of any type	1.91	(1.03–3.56)	0.043	1.89	(1.04–3.35)	0.038
GI disease	0.93	(0.50–1.74)	0.819	Not selected		
Liver disease	1.03	(0.42–2.57)	0.946	Not selected		
Falls in the previous year	1.13	(0.56–2.28)	0.735	Not selected		
Urinary incontinence	1.33	(0.68–2.61)	0.397	Not selected		
Chronic pain	1.21	(0.74–2.02)	0.444	Not selected		
Malnutrition	2.81	(1.34–5.96)	0.007	2.20	(1.05–6.17)	0.048
Polypharmacy	1.95	(1.14–3.36)	0.015	Not selected		
Cognitive impairment	2.18	(1.28–3.68)	0.004	1.97	(1.18–3.46)	0.010
Depression	2.17	(1.26–3.70)	0.005	Not selected		
BMI (kg/m^2^)	0.89	(0.82–0.98)	0.014	Not selected		
Gait speed (m/s)	0.67	(0.21–2.07)	0.486	Not selected		
Hemoglobin (g/L)	0.98	(0.97–0.99)	<0.001	Not selected		
Prealbumin (mg/L)	1.00	(0.99–1.01)	0.774	Not selected		

BMI: body mass index; CI: confidence interval; CKD: chronic kidney disease; COPD: chronic obstructive pulmonary disease; GI: gastrointestinal; HR: hazards ratio.

The multivariate COX proportional hazard analysis identified sarcopenia as an independent and significant predictor of 3-year all-cause mortality in the whole study population (HR: 2.06; 95% CI: 1.02–4.15). In addition, tumor of any type (HR: 1.89; 95% CI: 1.04–3.35), malnutrition (HR: 2.20; 95% CI: 1.05–6.17) and cognitive impairment (HR: 1.97; 95% CI: 1.18–3.46) were also significantly associated with an increased risk of 3-year all-cause mortality in the whole study population.

**Figure 2:**
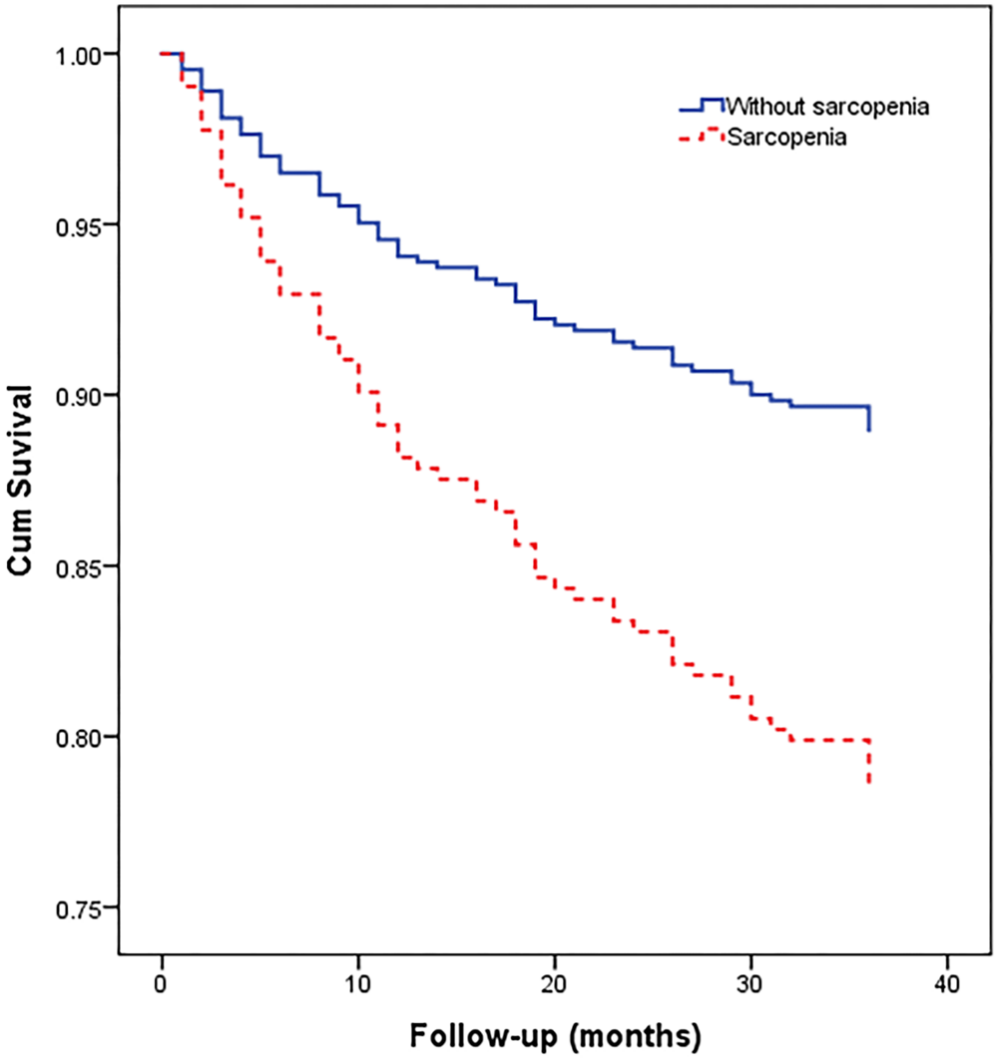
Survival curves for the whole study population according to sarcopenia status at baseline. Survival curves significantly differed in the log-rank test (p < 0.001).

The Kaplan-Meier survival curves for the subjects with or without sarcopenia during the 3-year follow-up period in the whole study population are presented in Fig. 2. The survival curves were significantly different by the log-rank test (P < 0.001).

**Figure 3:**
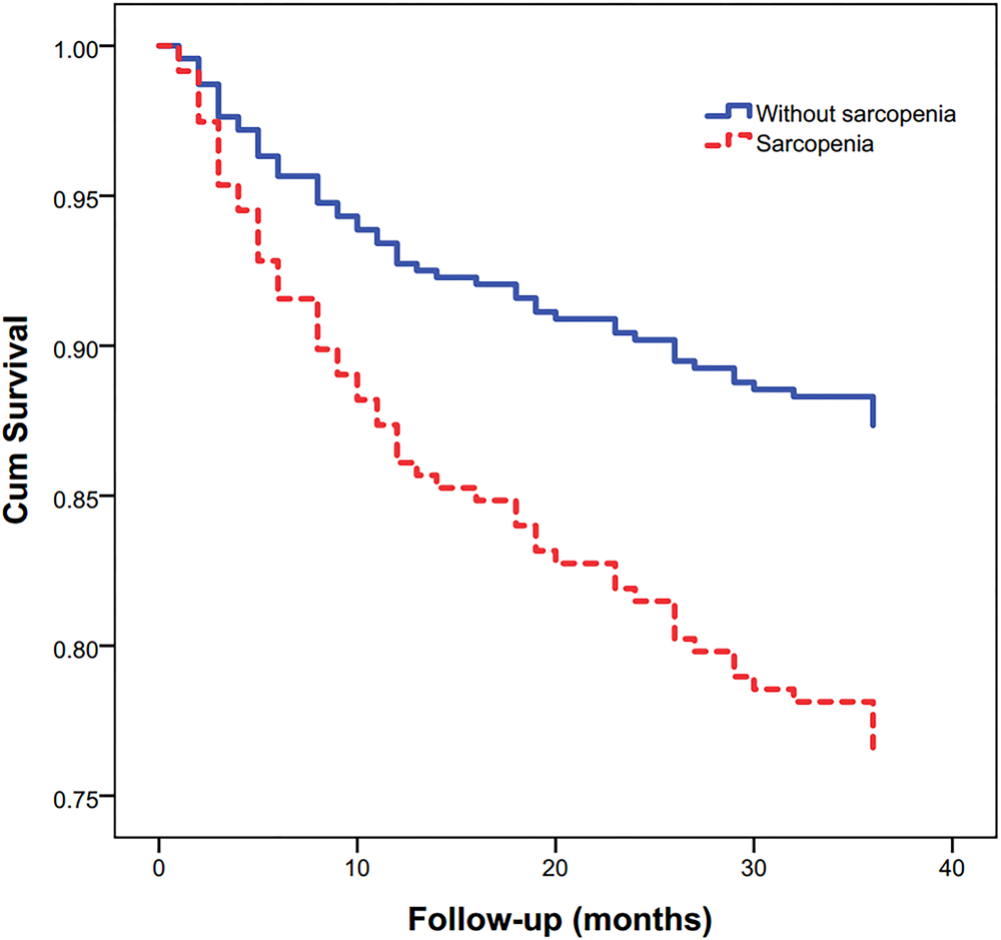
Survival curves for men according to sarcopenia status at baseline. Survival curves significantly differed in the log-rank test (p < 0.001).

**Figure 4:**
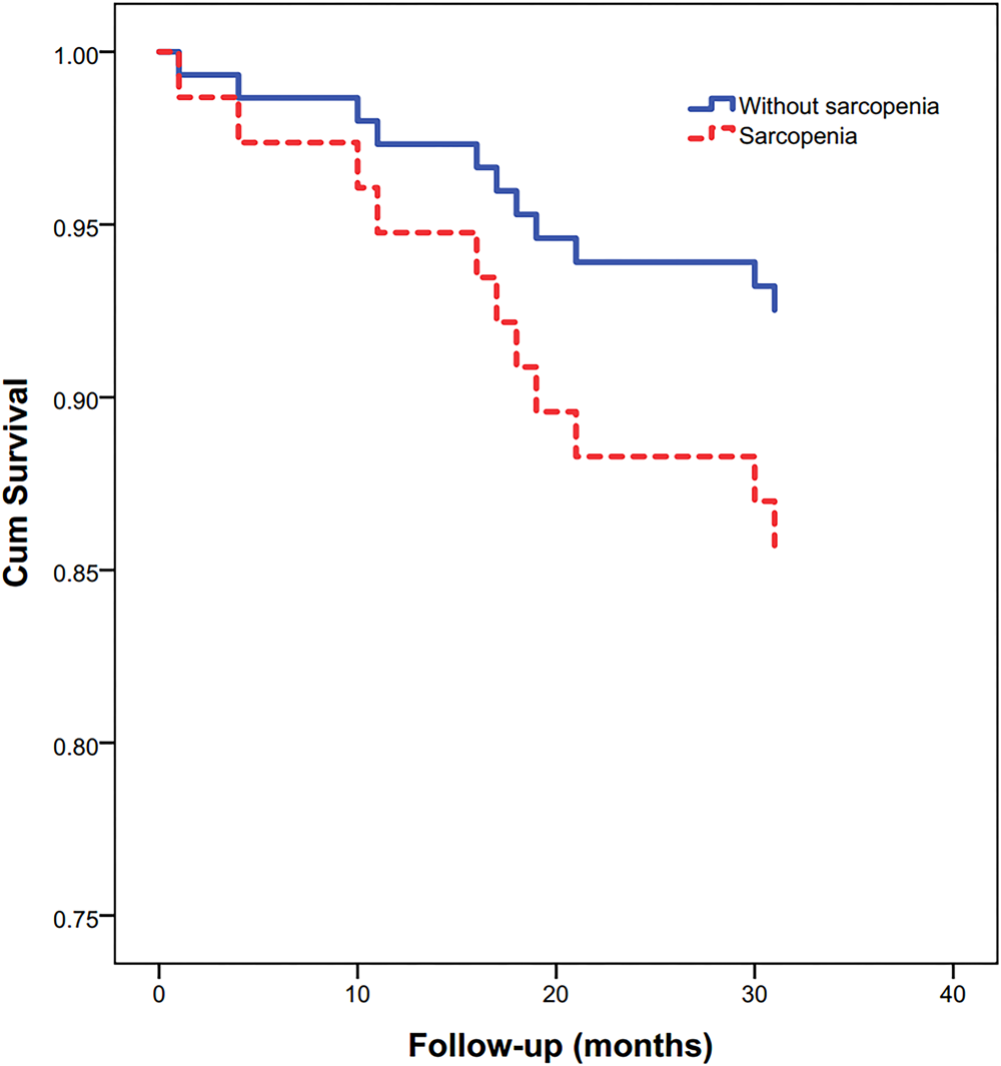
Survival curves for women according to sarcopenia status at baseline. Survival curves significantly differed in the log-rank test (p = 0.006).

The subgroup analyses found similar results in both men and women, however, gait speed was identified as an independent predictor for 3-year all-cause mortality in men but not in women (Table 3, Figs 3 and 4).

**Table 3:** Results of multivariate Cox proportional hazard analyses for 3-year all-cause mortality in men and women.

	Men			Women		
	HR	95% CI	p	HR	95% CI	p
Sarcopenia	2.40	(1.18–4.90)	0.016	1.85	(1.03–3.32)	0.039
Tumor of any type	1.84	(1.01–3.56)	0.045	1.31	(1.13–1.52)	<0.001
Malnutrition	1.24	(1.07–1.44)	0.004	2.12	(1.20–3.76)	0.009
Cognitive impairment	2.58	(1.46–4.57)	0.001	1.44	(1.08–1.91)	0.012
Gait speed (m/s)	0.87	(0.56–0.98)	0.035	Not selected		

CI: confidence interval; HR: hazards ratio.

## Discussion

To the best of our knowledge, this is the first study assessing the value of Ishii’s formula for predicting long-term all-cause mortality in hospitalized Chinese older adults. Using Ishii’s formula, the prevalence of sarcopenia was as high as 69.5% in our study population. Sarcopenia, defined by Ishii’s formula, was an independent predictor of long-term all-cause mortality.

There is currently no consensus on the diagnostic criteria for sarcopenia. However, several widely accepted criteria for sarcopenia have been developed by international organizations, such as the European Working Group on Sarcopenia in Older People (EWGSOP), the International Working Group on Sarcopenia (IWGS), the Asian Working Group for Sarcopenia (AWGS), and the Foundation for the National Institutes of Health (FNIH)^22^. All of these criteria recommended that sarcopenia should be defined by low muscle mass (measured with magnetic resonance imaging [MRI], computed tomography [CT], dual-energy X-ray absorptiometry [DXA], or bioelectrical impedance analysis [BIA]) and decreased muscle function (measured with GS and HS). However, MRI and CT are expensive, and CT and DXA increase the risk of radiation exposure. BIA is not routinely available in clinics and hospitals. Therefore, all current diagnostic methods for sarcopenia are not practical in clinical practice. Clinicians need a robust, practical, and implementable screening tool to identify patients who might suffer from sarcopenia.

As a pioneer in screening tools for sarcopenia, the SARC-F has been validated in previous studies^11,12,23^. The SARC-F is a self-report questionnaire including five domains: strength, ambulation, rising from a chair, stair climbing, and history of falls^11^. It can effectively exclude non-sarcopenic older subjects, hence avoiding unnecessary further assessment of those who are not at risk^23^. However, low sensitivity may result in missing people who are actually sarcopenic^24^. Ishii’s formula includes only three objective variables: age, HS, and CC^13^. This simple screening test has been approved by previous studies^25,26^. Our study supports the value of Ishii’s formula for predicting mortality in Chinese older adult inpatients. However, it is currently unclear which screening tool is better due to the lack of head-to-head comparative studies^24^.

The prevalence of sarcopenia in different clinical settings has been addressed in numerous previous studies. A recent systematic review of 35 articles reported that the prevalence of sarcopenia was 10% in community-dwelling participants when using the definitions of sarcopenia from the EWGSOP, IWGS, and AWGS^21^. Focusing on hospitalized older adults, previous studies reported that the prevalence of sarcopenia in acute geriatric wards was 25–28% when using the EWGSOP definition^27,28^. The prevalence of sarcopenia in our study was significantly higher (69.5%). This result was similar to a prospective study conducted among older adults in an in-hospital rehabilitation setting^25^. In that study, Morandi *et al*. defined “the probability of sarcopenia” using Ishii’s formula and reported that the prevalence of sarcopenia was 60%.

Our finding that sarcopenia was an independent predictor of long-term mortality in hospitalized older patients was consistent with previous studies^27,29,30^. For example, Vetrano *et al*. conducted a multi-center prospective study and found that sarcopenia, defined by the EWGSOP criteria using the BIA, HS, and GS, was associated with increased short-term and long-term mortality among hospitalized older adults^27^. Similarly, Cerri and colleagues reported that sarcopenia, defined by the EWGSOP criteria using the same method as Vatrano’s study, was associated with an increased risk of 3-month mortality in a population of malnourished hospitalized older adults or at those at risk for malnutrition^29^. Moreover, Gariballa *et al*. reported that sarcopenia, defined by the EWGSOP criteria using the mid-arm muscle circumference, HS, and GS, was also associated with 6-month mortality in hospitalized patients^30^. Putting this evidence together, it may be said that sarcopenia, no matter how it is measured, is an important factor for predicting mortality in hospitalized older adults.

Ishii’s formula has been validated among older patients in other clinical settings. For example, Yoshiro Onoue and colleagues^26^ evaluated the clinical utility of this formula in hospitalized Japanese older patients with heart failure in a prospective study. The results revealed that the sarcopenia score, calculated using Ishii’s formula, was an independent predictor of heart failure events. The authors concluded that Ishii’s formula can be used to predict adverse events in older patients with heart failure. In another prospective study, Alessandro Morandi *et al*.^25^ applied this formula in Caucasian older adults admitted to an in-hospital rehabilitation setting. They found that sarcopenia, defined by Ishii’s formula, was independently associated with poorer functional status and a less ability to walk at discharge. However, more prospective studies are needed to evaluate the validation of Ishii’s formula for diagnosing sarcopenia in different ethnic populations and clinical settings.

Not surprisingly, our study found that in both men and women, the gait speed was significantly slower in the sarcopenia group than in the non-sarcopenia group. In addition, previous studies demonstrated that gait speed is associated with mortality in older adults^31,32^. Our study also revealed the association between gait speed and mortality in men but not in women. The relatively small sample size of women compared to men in our study might partly explain this finding.

Some limitations of this study need to be addressed. First, Ishii’s formula was developed based on a Japanese older adult population. It is unclear whether this formula and the relevant cut-points are suitable for our study population. Considering the ethnic difference regarding HS and CC, modification of the formula and the cut-points may be required. However, Alessandro Morandi *et al*.^25^ successfully applied Ishii’s formula in Caucasian older adults. Combining these results, it could be argued that the value of Ishii’s formula for the screening of sarcopenia in clinical practice has been partly proven. Of course, there is still a long way to go before the generalizability of Ishii’s formula for the screening of sarcopenia is established. Second, in Ishii’s formula, CC is applied as a surrogate measure of muscle mass. Traditionally, anthropometric measures (e.g., CC) were not considered to be valid for estimating muscle mass^33^. However, recent prospective studies showed that the use of anthropometric measurements to estimate muscle mass combined with the HS and GS can predict mortality in older adults^34,35^. Third, we failed to adjust for some important confounders, such as activities of daily living and frailty. This may induce bias in our results. Forth, 27 (7.1%) participants lost to follow-up during the 3-year period. However, the baseline characteristics of those who completed the study and those who lost to follow-up were comparable. Therefore, our results were less likely to be biased by this issue.

## Conclusion

Sarcopenia, defined by a simple screening test (Ishii’s formula), is an independent and significant predictor of long-term all-cause mortality in a study population of hospitalized Chinese older adults. Ishii’s formula may be a valuable tool for screening sarcopenia in older adult inpatients. Further prospective studies with a larger sample size are needed to confirm the validity of this tool for predicting mortality and other important health outcomes (e.g., falls and quality of life) in different study populations.

## Electronic supplementary material

Supplementary Table 1. Baseline characteristics of participants according to follow-up status
