# Supplementary material for: A sarcopenia screening test predicts mortality in hospitalized older adults
Source: Sci Rep. 2018 Feb 13;8:2923. doi: 10.1038/s41598-018-21237-9 (PMC5811535; doi:10.1038/s41598-018-21237-9)
Supplement: Supplementary file 1 — Supplementary Table 1. Baseline characteristics of participants according to follow-up status [file 41598_2018_21237_MOESM1_ESM.pdf]

**Title page**

**Title:**

# A sarcopenia screening test predicts mortality in hospitalized older adults

**Authors:**

Tianjiao Tang [1], MD; Linna Wu [2], MSN; Ling Yang [3], MSN; Jiaojiao Jiang [4] PhD; Qiukui Hao [1], MD; Birong Dong [1,] MD; Ming Yang [1], MD

**Affiliations and addresses of all authors:**

1. The Center of Gerontology and Geriatrics, West China Hospital, Sichuan University, No.37 Guoxue Lane, Chengdu, Sichuan, China.
2. Health Management Center, West China Hospital, Sichuan University, No.37 Guoxue Lane, Chengdu, Sichuan, China.
3. Outpatient Department, West China Hospital, Sichuan University, No.37 Guoxue Lane, Chengdu, Sichuan, China.
4. The Center of Rehabilitation, West China Hospital, Sichuan University, No.37 Guoxue Lane, Chengdu, Sichuan, China.

**Information of the corresponding author:**

Ming Yang, MD, The Center of Gerontology and Geriatrics, West China Hospital of Sichuan University, 37 Guoxue Lane, Chengdu, China. Phone: +86 28 8542 2326. Fax: +86 28 8542 2321. Email: yangmier@gmail.com

**Supplementary Table 1. Baseline characteristics of participants according to follow-up status**

| Characteristic | Completed follow-up (n=353) | Loss of follow-up (n=27) | p |
|---|---|---|---|
| Age (years) | 80.4 ± 7.0 | 77.7 ± 9.4 | 0.058 |
| Women | 89 (25.2) | 11 (40.7) | 0.077 |
| Current smokers | 42 (11.9) | 4 (14.8) | 0.654 |
| Current alcohol drinkers | 44 (12.5) | 5 (18.5) | 0.366 |
| Sarcopenia | 244 (69.1) | 20 (74.1) | 0.590 |
| Comorbidities | | | |
| Hypertension | 211 (59.8) | 14 (51.9) | 0.420 |
| Ischemic heart disease | 116 (32.9) | 7 (25.9) | 0.458 |
| COPD | 110 (31.2) | 9 (33.3) | 0.815 |
| Diabetes | 96 (27.2) | 8 (29.6) | 0.785 |
| Stroke | 21 (5.9) | 4 (14.8) | 0.073 |
| CKD | 50 (14.2) | 3 (11.1) | 0.659 |
| Acute infection | 107 (30.3) | 8 (29.6) | 0.941 |
| Osteoarthritis | 96 (27.2) | 7 (25.9) | 0.886 |
| Tumor of any type | 41 (11.6) | 1 (3.7) | 0.206 |
| GI disease | 68 (19.3) | 5 (18.5) | 0.925 |
| Liver disease | 27 (7.6) | 4 (14.8) | 0.190 |
| Falls in the previous year | 42 (11.9) | 4 (14.8) | 0.654 |
| Urinary incontinence | 42 (11.9) | 6 (22.2) | 0.120 |
| Chronic pain | 108 (30.6) | 11 (40.7) | 0.273 |
| Malnutrition | 35 (9.9) | 5 (18.5) | 0.150 |
| Polypharmacy* | 127 (47.6) | 1 (16.7) | 0.134 |
| Cognitive impairment* | 94 (35.2) | 2 (33.3) | 0.924 |
| Depression* | 67 (25.1) | 1 (16.7) | 0.637 |
| BMI (kg/m$^2$) | | | |
| Women | 22.9 ± 4.4 | 21.6 ± 2.7 | 0.350 |
| Men | 22.4 ± 3.7 | 21.0 ± 3.1 | 0.158 |
| CC (cm) | | | |
| Women | 31.7 ± 4.4 | 31.3 ± 3.8 | 0.758 |
| Men | 32.8 ± 3.8 | 31.3 ± 3.6 | 0.122 |
| Gait speed (m/s) | | | |
| Women | 0.7 ± 0.3 | 0.8 ± 0.1 | 0.763 |
| Men | 0.8 ± 0.4 | 0.8 ± 0.4 | 0.833 |
| Handgrip strength (kg) | | | |
| Women | 14.1 ± 6.9 | 13.4 ± 7.7 | 0.747 |
| Men | 22.3 ± 8.7 | 19.8 ± 6.1 | 0.271 |
| Hemoglobin (g/L) | 122.6 ± 22.4 | 121.7 ± 21.8 | 0.857 |
| Prealbumin (mg/L) | 195.0 ± 62.3 | 197.2 ± 61.3 | 0.873 |

* The sample size was 273 due to missing data.

Data are presented as the number (percent) for the following variables: women, current

smokers, current alcohol drinkers, and specific comorbidities listed above. For other variables, the mean ± SD are presented.

One-way ANOVA was used for the continuous variables, and the Pearson chi-squared test was used for categorical variables. During analyses, $p < 0.05$ was considered statistically significant.

BMI: body mass index; CC: calf circumference; CKD: chronic kidney disease; COPD: chronic obstructive pulmonary disease; GI: gastrointestinal
